# Novel approaches for the design, delivery and administration of vaccine technologies

**DOI:** 10.1111/cei.13287

**Published:** 2019-04-08

**Authors:** J. Wallis, D. P. Shenton, R. C. Carlisle

**Affiliations:** ^1^ Institute of Biomedical Engineering University of Oxford Oxford UK; ^2^ Defence Science and Technology Laboratory Porton Down UK

**Keywords:** delivery, novel, vaccine, vaccination

## Abstract

It is easy to argue that vaccine development represents humankind’s most important and successful endeavour, such is the impact that vaccination has had on human morbidity and mortality over the last 200 years. During this time the original method of Jenner and Pasteur, i.e. that of injecting live‐attenuated or inactivated pathogens, has been developed and supplemented with a wide range of alternative approaches which are now in clinical use or under development. These next‐generation technologies have been designed to produce a vaccine that has the effectiveness of the original live‐attenuated and inactivated vaccines, but without the associated risks and limitations. Indeed, the method of development has undoubtedly moved away from Pasteur’s three Is paradigm (isolate, inactivate, inject) towards an approach of rational design, made possible by improved knowledge of the pathogen–host interaction and the mechanisms of the immune system. These novel vaccines have explored methods for targeted delivery of antigenic material, as well as for the control of release profiles, so that dosing regimens can be matched to the time‐lines of immune system stimulation and the realities of health‐care delivery in dispersed populations. The methods by which vaccines are administered are also the subject of intense research in the hope that needle and syringe dosing, with all its associated issues regarding risk of injury, cross‐infection and patient compliance, can be replaced. This review provides a detailed overview of new vaccine vectors as well as information pertaining to the novel delivery platforms under development.

## Introduction

Vaccines are arguably the most important medical technology developed to date, and have provided dramatic reductions in disease morbidity and mortality since Edward Jenner first tested his smallpox vaccine in 1798 1. The World Health Organization (WHO) estimates that vaccinations for diphtheria, tetanus, whooping cough and measles currently prevent 2–3 million deaths per year 2. Smallpox was once one of the most feared diseases, until the implementation of global vaccination programmes enabled it to be declared eradicated in 1979 3. This eradication cost approximately 100 million US dollars ($US), but is estimated to generate annual savings of 1.35 billion $US 4.

Although vaccines have been undeniably successful, improvements in vector production, delivery and ease of use would be of great benefit. Historically, vaccine development has been based on the ‘three Is’ paradigm of Louis Pasteur (isolate, inactivate, inject) 5. However, an improved understanding of immunology, pathology and microbiology is now helping vaccine development to adopt a more ‘rational design’ approach 5, 6. A large portion of these rationally designed vaccines consist of a ‘minimalist’ composition (i.e. they are subunit‐ or peptide‐based), and while this provides safety and cost‐of‐production benefits, they are typically less immunogenic 7. However, it is hoped that the optimization and combination of rational design approaches and the use of novel dosing and adjuvanting strategies can help to close this efficacy gap.

Delivery is an important issue, because the majority of vaccines used currently are still administered with a hypodermic needle, either intramuscularly (e.g. hepatitis B or inactivated poliomyelitis), subcutaneously (e.g. measles or yellow fever) or intradermally [e.g. bacillus Calmette–Guérin (BCG)]. The hypodermic needle is the mainstay of vaccine delivery technology because it provides a direct, low‐cost method of administration with instant validation that the dose has been delivered and an impressive efficacy profile defined over decades of use. However, the many drawbacks and limitations of needle and syringe delivery are beginning to make it look like a rather out‐dated approach. Prime among these limitations is the effect pain and fear of needles has on patient compliance and ultimately vaccination rates 8. In the United Kingdom and United States, infants may have received up to 23 prophylactic inoculations for 10 different pathogens by the age of 18 months 9. A further major concern, for developed but especially developing countries, is the spread of blood‐borne pathogens as a consequence of needle reuse or needle‐stick injuries 10, 11. In 2000, approximately 16 billion injections were administered, of which an alarming 40% were administered with reused equipment in the absence of sterilization 12. This led to an estimated 20·6 million new hepatitis B infections, 2 million new hepatitis C infections and 260 000 new HIV infections 12. In 2000, approximately half of all US physicians and 77% of nurses experienced needle‐stick injuries, which led to 16 000 hepatitis C, 66 000 hepatitis B and 1000 HIV infections 13, 14. It is clear that in the intervening 18 years improved training and working practices have reduced these levels, but a more recent report still showed that 14·9–69·4% of health‐care workers have reported needle‐stick injuries, with the wide range due to differences in practices between countries 15. This report also showed that needle‐stick injuries were responsible for 37–39% of global hepatitis B and C infections in health‐care workers 15. It is no surprise that when faced with this iatrogenicity, researchers have looked to develop alternative approaches that might allow vaccination without the use of a needle. A further limitation of the reliance on needles is the requirement it creates for the use of liquid formulations, which in many cases require expensive cold‐chain transport and storage 16.

This review begins by giving a broad overview of novel approaches in vaccine design and composition and describes how formulation approaches are improving delivery platforms (see below: ‘Novel vaccine designs’ and ‘Novel vaccine delivery platforms’). A further notable means of improving vaccine efficacy, the development and use of technologies for the improved administration of vaccines, is then covered in the later section (see below: ‘Vaccine administration routes and technologies’).

## Novel vaccine designs

### Virus‐like particles

Virus‐like particles (VLPs) are highly ordered, repetitive structures that contain a high density of viral capsid proteins. This high density of capsid proteins provides copious amounts of conformational viral epitopes, capable of eliciting strong immune responses 17. Crucially, VLPs are formed by the self‐assembly of viral capsid proteins in the absence of any of the infectious nucleic acids from the virus. Thus, they are a potentially safer alternative to the attenuated viruses commonly used for vaccination due to their absolute inability to replicate. VLPs have been shown to be capable of generating strong immune responses, even in the absence of an adjuvant 18.

Being simpler in composition, VLPs also allow faster production of vaccine than traditional methods, which is especially useful for treatment of highly mutating pathogens such as influenza. Traditional production of an influenza vaccine takes 9 months after a new annual strain has been sequenced, but VLP production takes only 3–12 weeks 19, 20. The first VLP vaccine to be brought to market was the vaccine for hepatitis B (Recombivax HB), in 1986, which consists of self‐assembled particles made from the virus capsid protein, hepatitis B surface antigen 21. Since then, VLP vaccines for human papillomavirus (HPV) (Gardasil) and hepatitis E (Hecolin) have also made it to market in 2006 and 2011, respectively 22, 23, with many more undergoing evaluation in clinical trials 24.

### Conjugate vaccines

Vaccines that use either live‐attenuated or inactivated pathogens contain a wide array of different antigens, both polysaccharide‐ and protein‐based. However, only a small number of these may be required to induce protective immunity 25, 26. This logic has been further extended for proteins by the realization that each protein contains hundreds of possible immunogenic epitopes, not all of which are necessary. This has led to interest in peptide‐based vaccines 25. However, antigenic epitopes on a protein are not simply a sequence of amino acids, as the peptides used must mimic the conformation of the immunogenic epitope within the native protein. Computational modelling has provided a powerful tool for locating and mapping the conformation of immunogenic epitopes within proteins 27, 28.

**Figure 1:**
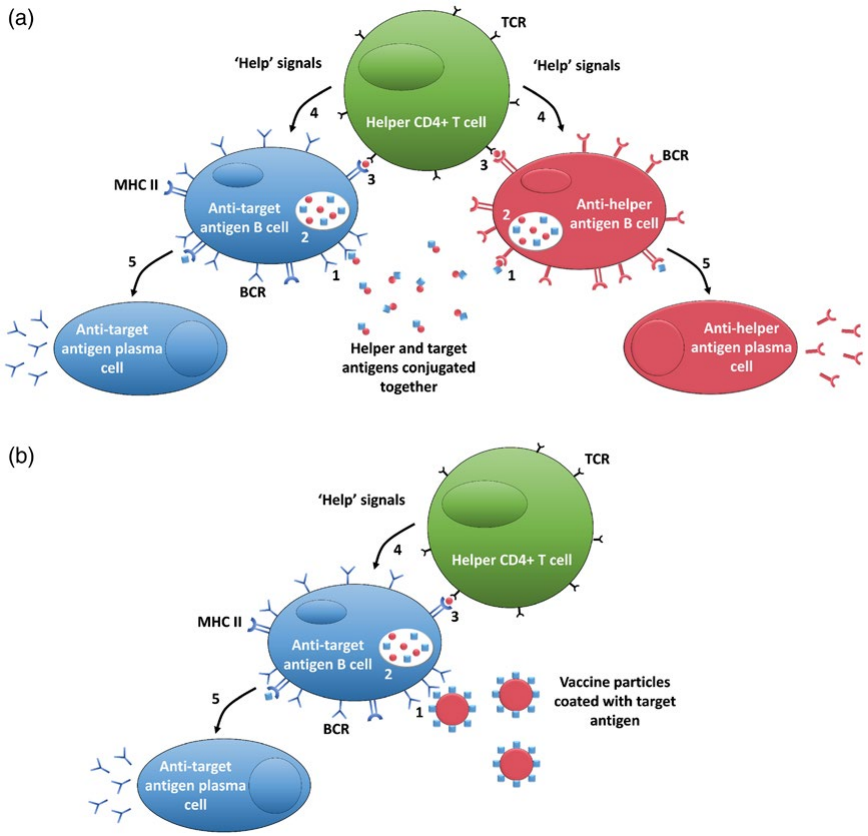
Mechanism of action for initiation of humoral immune responses to a target antigen aided by a secondary helper antigen when they are conjugated together (a) and spatially segregated by use of a liposome (b). (1) B cell with specific B cell receptor (BCR) for target/helper antigen binds to vaccine particle; (2) B cells engulf and digest vaccine particles; (3) vaccine particle antigens are presented on B cells class II major histocompatibility complexes (MHC II) for helper T cell recognition with specific T cell receptor (TCR) for presented antigen; (4) upon TCR binding, T cells produce ‘help’ signals to stimulate B cell differentiation; (5) B cells differentiate into plasma cells that secrete antibodies exclusively targeting antigen recognized by BCR in step 1.

Peptide‐ or polysaccharide‐based vaccines tend to be less immunogenic than when they are present on the surface of a pathogen, thus they require the inclusion of an adjuvant when being administered 7, 29. Another possibility is to conjugate the antigen to a second ‘helper’ protein or polysaccharide that is known to increase immunogenicity; however, this can lead to the immune response being redirected towards the helper molecule 26, 30, 31. Careful pairing and orientation of target and helper portions of the vaccine or spatial segregation of the two subunits by use of carrier systems, such as liposomes, are approaches to overcome this issue (Fig. 1) 32, 33. Peptides and polysaccharides are relatively cheap and simple to manufacture synthetically, which also removes the risk of contamination with infectious material, as is possible with traditional live‐attenuated or inactivated vaccines. Conjugate vaccines for haemophilus influenzae type B (Hib), pneumococcus (PCV), meningococcus (MenACWY) and malaria (Mosquirix) have been approved for use in humans. The RTS,S/AS01 (Mosquirix) vaccine, in particular, is a conjugate vaccine of a repeated region of the circumsporozoite protein from *Plasmodium* sporozoites conjugated to the hepatitis B surface antigen. This conjugate vaccine subsequently assembles into a VLP.

### Nucleic acid vaccines

Long‐term gene expression from plasmid DNA has been demonstrated to be achieved by intramuscular injection in mice 34. Such plasmids can be used to encode a viral antigen, which can lead to both humoral and cellular antigen‐specific immune responses 35. These studies led to a huge amount of research into DNA‐based vaccines against a multitude of diseases, such as influenza, human immunodeficiency virus (HIV) and lymphocytic choriomeningitis virus (LCMV) 36, 37, 38. Practically, DNA vaccines are more cost‐effective than protein, whole cell or viral vectors, as DNA can be synthesized by simple scalable chemistry or produced at scale in bacteria. However, the main drawback of DNA vaccines is their low immunogenicity due to the very low transfection rate they achieve. Non‐condensed plasmid is a highly distended and negatively charged structure, which is prone to degradation in the extracellular compartment and lacks a mechanism to achieve cell entry. Even if cell entry can be achieved, localization to and entry into the nucleus, which are required to achieve transcription, are extremely inefficient 39. To increase immunogenicity, plasmids can be designed to encode multiple antigens as well as other immunostimulatory molecules to induce an adjuvanted immune response 40. While no DNA vaccine has yet been licensed for use in humans, there have been several licensed for veterinary use, including West Nile (West‐Nile Innovator DNA) and salmon pancreas disease (Clynav) 41, 42.

The limitations of DNA vectors have resulted in RNA‐based vaccines gaining momentum in recent years 43. Like DNA‐based vaccines, they are low‐cost and can be manufactured rapidly on a large scale. However, their application has previously been restricted by the instability of RNA and inefficient *in‐vivo *delivery 43. Several methods of structural modification have been employed to increase the intracellular stability of the RNA molecules 44. Crucially, in contrast to DNA, RNA does not require targeting to and entry into the nucleus, so the main barrier RNA vaccines face is cell entry 45. This can be addressed by formulation with polycationic carrier molecules that can condense and protect the RNA and aid its rapid cellular uptake 46.

The main focus of RNA‐based vaccine development has been cancer, with numerous Phases I–III clinical trials in progress 43, 47. For infectious pathogens, two major types of RNA vaccine have been utilized: non‐replicating and self‐amplifying. Non‐replicating RNA vaccines are simpler and less expensive to manufacture, but may be limited in the duration and level of expression they can achieve. Self‐amplifying RNA systems can be based on sequences and principles borrowed from single‐positive strand RNA viruses, such as alphaviruses (Alphavax). These vectors encode the non‐structural genes and the immunogen, but no structural genes, so in theory can achieve a single replication cycle without the risk of infectious virus production. They therefore enable a small dose of vaccine to produce a large amount of antigen due to intracellular amplification of the antigen‐encoding RNA. Several clinical trials using RNA‐based vaccination have been undertaken for infectious pathogens such as HIV, rabies and zika 48, 49, 50. While RNA may currently seem to be the more attractive of the nucleotide‐based options, it should be noted that DNA potentially provides advantages in terms of coding capacity and the level and duration of immunogenic protein production. Should the delivery barrier faced by DNA be overcome, a resurgence in the interest in its use may follow. The recent development of scalable, cell‐free, enzyme‐driven DNA production technologies strengthens the case for the translatability of DNA vaccines 51.

### Cellular vaccines

Due to the history of success of vaccination using live‐attenuated viruses, inactivated viruses or bacteria, attempts have been made to apply a similar approach to vaccinate against cancer. Attenuated tumour cells have been administered to induce an immune response against specific types of cancers. Two types of whole cell vaccines have been used: autologous and allogenic. Autologous cell vaccines have been tested on a variety of cancers, including lung, colorectal, melanoma, renal and prostate cancer 52, 53, 54, 55, 56. However, autologous cell vaccines are limited to only certain types and stages of tumours, as they require a sufficient amount of the patient’s tumour for preparation. In contrast, allogenic cell vaccines typically contain a combination of established human tumour cell lines and so, while not being patient‐specific, they do not have the production limitations of autologous cell vaccines. Many whole cell vaccines have been genetically modified to increase immune stimulation by inducing expression of cytokines, chemokines and co‐stimulatory molecules 55, 57, 58. To date, their clinical impact has been demonstrated by GVAX, a vaccine for the treatment of pancreatic cancer, currently in Phase II clinical trials 59.

Another kind of cellular‐based vaccination exploits a patient’s own immune cells, specifically their dendritic cells. Dendritic cell vaccines can be formulated by loading a patient’s autologous dendritic cells, that are simultaneously treated with immunoadjuvants with either tumour‐associated antigens or nucleic material encoding for tumour‐associated antigens *ex vivo*. The newly matured, antigen‐loaded dendritic cells are then readministered to the patient to induce anti‐tumour immunity. Dendritic cell vaccines have been tested against prostate, melanoma, renal and glioma tumours in clinical trials 60, 61, 62, 63. The first therapeutic cancer vaccine to receive Food and Drug Administration (FDA) approval was a dendritic cell‐based vaccine for prostate cancer, Sipuleucel‐T (Provenge) 64. This was on the basis of a Phase III trial in 2010, which found that patients receiving Sipuleucel‐T had a 4.1‐month median improvement in overall survival 65, 66. This approval was a great boon for this approach, but the development and approval rate of similar strategies has since been unremarkable. This may reflect factors relating to intellectual property or may be a consequence of the expense of the treatment ($93 000 for three infusions of the treatment). This vaccine regimen requires the isolation of peripheral blood mononuclear cells from the patient, followed by cell culture processing and reinfusion, each of which is a costly and laborious process. Indeed, while these cell‐based approaches are an interesting development, they do not obviously contribute to the continuum of the journey away from live and attenuated vaccines into an era of vaccines with lower complexity and cost of production that are more suited to the treatment of large populations within the confines of reducing health‐care resources.

From this section, it is clear that there is a range of novel vaccines with impressive immunological activity. It is notable that the majority of these have been designed and are being used under the presumption that conventional needle‐and‐syringe delivery offers the best route to their optimal efficacy. The following sections provide information on vaccine formulation delivery platforms and administration routes/technologies which may be worthy of consideration as a means to challenge this dogma and perhaps enhance vaccine utility further.

## Novel vaccine delivery platforms

### Liposomes

Liposomes are spherical vesicles with a lipid bilayer formed of biocompatible phospholipids. Their main use in vaccinology is either as a delivery vehicle or as an adjuvant 67, 68. A key advantage of liposomes is their plasticity and versatility; the choice of lipids and their formulation method allows control over their charge, size and location of antigen incorporation 69. Liposomes are often made up of four key components: a charged lipid, which affects how liposomes behave *in vivo*; a lipid‐linked polyethylene glycol (PEG), to increase *in‐vivo *stability; cholesterol, to increase structural stability; and a phospholipid, which supports the formation of a lipid bilayer. Cationic liposomes, unlike their anionic counterparts, are able to bypass the endosomal–lysomal route of degradation in cells 70. Furthermore, their net positive charge provides a means of condensing nucleic acid constructs (DNA or RNA) into discrete structures capable of achieving entry into target cells 70.

Antigens can be encapsulated within, conjugated to the surface of, or embedded within the lipid bilayer 33, 71. The location of antigen in liposomes influences the type of immune responses generated towards the vaccine. T cell responses are induced by both encapsulated and surface‐conjugated antigens, while B cell responses are exclusively induced by surface‐conjugated antigen 67. Incorporation of CD4 T cell helper epitopes can aid in generating a stronger antibody response to a B cell target antigen 33, 72, and complete spatial segregation of the two antigens by the liposomal bilayer minimizes the immune responses focus on the T cell epitope (Fig. 1) 33. The carrying capacity of liposomes allows immunostimulatory molecules, such as cytokines or Toll‐like receptor agonists, to be co‐delivered to target immune cells, thereby reducing systemic exposure to these adjuvant molecules 33, 67. Liposomes were first used as part of a vaccine for diphtheria toxin in 1974 73. Since then, liposome‐based vaccines for hepatitis A (Epaxal) and influenza (Inflexal V) have been approved for use in humans 74, 75.

### Polymeric particles

Polymeric particles have been increasingly researched in the field of vaccine delivery due to their potentially advantageous biocompatibility and biodegradability 76. A wide range of both natural and synthetic polymers has been used to make particles for vaccine delivery, such as polysaccharides 77, poly(D,L‐lactic‐coglycolic acid) (PLGA) 78, poly(lactic acid) (PLA) 78 and poly(D,L‐lactide‐co‐glycolide) (PLG) 79. These particles are able to either entrap or adsorb antigen for delivery to specific cells or allow for sustained antigen release over time because of their slow biodegradation rate 80, 81. Polymeric particles are the main focus for development of a single‐dose delayed‐release vaccine that could replace the need for booster doses of many current vaccines 81. The antigen release profile of a PLGA particle can be modified from a couple of days to more than a year 82, 83. Many studies have also used polymeric particles for their ability to act as an adjuvant, rather than their antigen release profile. Advax, an insulin‐derived microparticle, has been used in clinical trials as an adjuvant for hepatitis B, influenza and insect‐sting allergy vaccines 84, 85, 86.

### Inorganic particles

Many inorganic particle‐based vaccines have been studied, despite their low biodegradability. Their main advantage lies in how much control can be achieved over their synthesis 87, 88. Inorganic particles have been used as both adjuvants and antigen delivery vehicles in order to enhance an immune response 89. The four most commonly used types of inorganic particles deployed in vaccines are: gold, aluminium, calcium phosphate and silica. Structures made from pure carbon have also been investigated.

Gold particles are highly stable and can be easily synthesized in a variety of different shapes and sizes 87. Their surface is highly modifiable, making antigen conjugation straightforward in practice 90, 91. However, there is often limited control over the orientation of antigen, which can be suboptimal 92. Gold particles have been used as carriers in several clinical trials for a range of diseases, including melanoma, influenza and hepatitis B 93, 94, 95.

Aluminium is a commonly used adjuvant in vaccines 88, 96, 97, 98. This stimulated some studies into the conjugation of antigen to aluminium nanoparticles 97, 98. These studies showed that the aluminium particles are able to play the role of both carrier and adjuvant to stimulate the immune system, although it has also been shown that aluminium particles are capable of adsorbing antigen so tightly that antigen structure is altered, which reduces vaccine efficacy 98, 99.

Calcium phosphate particles are a promising candidate for vaccine applications as they are bioresorbable, non‐toxic, have adjuvanting properties and can easily be loaded with antigen 100. Previously, calcium phosphate was used as an adjuvant for a commercialized diphtheria, tetanus, pertussis and poliomyelitis vaccine, but was replaced by aluminium salts in the 1980s 101. It has been suggested that calcium phosphate should replace aluminium as an adjuvant in currently commercialized vaccines due to toxicity and side‐effect concerns with aluminium adjuvants 92, 101, 102.

Silica‐based particles are a popular form of inorganic particle in vaccine research, as they are biocompatible and their interactions with cells can be modified by altering their size and shape 103. Their surface can also be modified to allow for improved cellular targeting and cellular uptake 104, 105. Mesoporus silica particles have been shown to be effective antigen carriers for sustained antigen *in vivo* 106.

Carbon nanoparticles have also been studied extensively for drug and vaccine delivery 76. They can be synthesized into a variety of different nanotubes and mesoporous spheres 107, 108. Carbon nanotubes are potentially capable of carrying multiple antigens and are rapidly taken up by antigen‐presenting cells 109. Carbon nanoparticles have also been synthesized to be responsive to magnetic force and, with a model antigen attached, were used to track, target and manipulate dendritic cells 110. Carbon mesoporous spheres, encapsulating antigen, have been utilized as an oral vaccination method for bovine serum albumin as a model antigen 111.

### Plant‐like material

Plant cells are an attractive vaccine delivery platform for oral administration because of the ease and low cost with which large populations could be vaccinated. Furthermore, their tough cell wall is able to protect intracellular material from harsh environments encountered within the stomach 112. Once within the gastrointestinal tract, the cell wall is then broken down by microbes and the intracellular material is released 112. Transgenic plant cells have been produced to express antigenic material for use in vaccination 113. These transgenic cells may then be able to deliver antigenic material to the intestines, where it is free to interact with the gut‐associated lymphoid tissue. Crops such as rice and maize have been utilized as expression vectors. These crops are staple foods in target vaccination areas, are inexpensive to produce and easy to grow in large quantities 114, 115. Plant cell‐based vaccines have been developed for a wide range of pathogens, including influenza, hepatitis B and anthrax 115, 116, 117. A potential limitation of this approach is how the complexity and diversity of the microbiome will impact upon the reliability of the response.

Similar to plant cells, single‐cellular algae also have a tough cell wall, making them another attractive delivery platform. Algae‐based vaccines have many advantages over plant cell‐based vaccines: they are easier to genetically modify, they can be grown in bioreactors and do not require large areas of land, seasonal conditions or extended durations to grow 118. Several pathogens have been targeted preclinically by algae‐based vaccines such as foot‐and‐mouth disease, malaria and staphylococcus 119, 120, 121.

Pollen grains are naturally occurring plant‐based materials that have also recently been investigated as a vaccine delivery platform 122. They consist of a tough outer shell that is used to protect the male gamete of the plant for pollination. The outer shell has been shown to be able to survive the harsh conditions of the stomach, thus making them a possible delivery platform for oral vaccination 123. Recently, it has been shown that *Lycopodium clavatum *(clubmoss) and *Ambrosia elatior *(ragweed) spores can be chemically cleaned to remove any native proteins present that risk being allergenic, before being refilled with proteins of interest for vaccination 122, 124. These studies demonstrated that systemic and mucosal antibody responses could be generated against the model antigen, ovalbumin, when encapsulated within the pollen grains. Questions remain concerning the complexity and scalability of this approach.

### Infectious material

Bacteria and viruses can be genetically modified to produce antigenic material from another pathogen 125, 126. The bacterial or viral strains used for this method of vaccination are generally considered to be safe, either through natural lack of pathogenicity or through attenuation, but can still closely mimic a natural infection, and therefore can stimulate an immune response. However, the immune response generated is often dominated by a response against the carrier vector and not the desired target antigen 127.

### Outer membrane vesicles

Outer membrane vesicles are naturally occurring, non‐replicating vesicles produced by Gram‐negative bacteria 128. They consist of bacterial phospholipids, lipopolysaccharides, outer membrane proteins and entrapped periplasmic components 128, 129. This gives them inherent immunostimulatory properties, as they naturally contain several pathogen‐associated molecular patterns on their surface, which makes them an adjuvanting particle. Antigen can be present either on the surface, inside the lumen of the vesicle or unbound in solution. Antigens within the lumen of the vesicles were believed to be hidden from B cell recognition. However, several groups have reported strong antibody responses to a luminal antigen 130, 131. One of the difficulties of using outer membrane vesicles to target an antigen non‐native to the producing bacteria is that the vesicles naturally contain many immunogenic components, which could lead to an immune response dominantly targeting the vesicle instead of the antigen of interest. To date, two outer membrane vesicle vaccines for meningitis B (Bexsero and Trumenba) have been licensed for use in humans 132, 133.

### Immunostimulating complexes

Immunostimulating complexes (ISCOMs) are spherical cage‐like particles that are spontaneously formed by mixing phospholipids, cholesterol, saponin and protein antigens 134, 135. ISCOM formulation requires the use of amphipathic proteins which restricts the type of antigens that can be included in the complex 136. An alternative form of ISCOM is ISCOMATRIX, which is formulated without antigen 136. This approach allows for a more flexible application, as almost any antigen can potentially be mixed with the ISCOMATRIX adjuvant. ISCOMATRIX adjuvant has been used for both prophylactic and therapeutic vaccines in clinical trials 137, 138.

### Emulsions

Emulsions are heterogeneous liquid systems commonly used in vaccines as adjuvants. Their simplest iteration is in the form of water‐in‐oil or oil‐in‐water, but they can be formulated in more complex multiple emulsion systems such as water‐in‐oil‐in‐water 139. The antigen release characteristics of an emulsion are determined by a range of factors such as droplet size, viscosity and the oil‐to‐water ratio 140. One emulsion frequently used in human vaccines today is an oil‐in‐water, squalene‐based emulsion, MF59, which has been included in the inactivated flu vaccine, Flaud, since 1997 140, 141.

## Vaccine administration routes and technologies

### Transdermal

Using a needle and syringe is a very effective method of introducing a substance to the body, as the barrier properties of the skin are very easily breached by a needle. However, use of needles and syringes has many disadvantages, such as pain, needle phobia, risk of needle‐stick injuries and transmission of infections, all of which lead to increased cost and poor patient compliance 8, 12. Therefore, there is a great need for alternative methods of vaccination that do not have these disadvantages. The skin houses a large number of immune cells indicating that the skin is a hub of immunological activity, and a target location for vaccine administration 142. Indeed, when compared to traditional vaccination methods using a needle and syringe, transdermal delivery has been shown to be capable of inducing improved immune responses 143.

Some studies have investigated the use of passive delivery methods to administer vaccines transdermally 144, 145. These efforts have focused on several drugs that are already licensed for transdermal administration, such as nicotine and testosterone 145. However, in order to be amenable to successful passive delivery, vaccine molecules must have a low molecular weight, be reasonably lipophilic and have a very high potency (as the percentage of dose delivered is so low) 145. The main drawback of passive delivery is the long lag time to induce a response 146; this has been demonstrated by one study that showed a prolonged exposure of 16 h to antigen on the skin was needed to induce a potent antigen‐specific response 144.

Arguably, the most explored method of transdermal delivery is in the use of microneedles, which consist of 10s to 1000s of pointed microsized projections fabricated onto a surface 147. There have been a number of different microneedle systems developed, including: solid 148, hollow 148, coated 149 and dissolving 150. Solid microneedles have been fabricated from a range of materials such as silicon, polymers, water‐soluble compounds, metals and ceramics 147 and can be used to permeabilize the skin before topical application of the vaccince 148. Hollow microneedles are similar to hypodermic needles in that they enable pressure‐driven injection of a liquid allowing control over injection rate, although they have also been used to deliver drug reservoirs without the use of a pressure force 147, 148. Coated microneedles use solid microneedles as vehicles to deliver drug or vaccine deposited on their surface into the dermal layers; this may be a quick method to administer the desired dose 147, 149. Dissolving microneedles are an ideal alternative to hypodermic needles, as they are designed to completely dissolve when inserted into the skin, therefore leaving no hazardous sharps waste 147, 150. Microneedles have been used for both transdermal and mucosal vaccination 147, 149. They can be self‐administered without the need for professional training, thus easing the burden on medical staff 147. It is theorized that as microneedles are so small, they are unable to penetrate deep enough into the skin to cause pain, therefore potentially increasing patient compliance 147. To date, no microneedle technology has been FDA‐approved for the delivery of a vaccine. Important remaining barriers to such translation include skin irritation, confirmation of dose delivered, scale‐up and compatibility of vaccines with the microneedle manufacture process. As microneedles contain many microscopic needles, they may not ultimately reduce the amount of medical sharps waste currently generated.

Electricity has also been used to facilitate the delivery of drugs and vaccines transdermally in two differing methods, iontophoresis and electroporation. Iontophoresis relies on the application of an electrical current to drive charged particles into the skin through electrostatic effects 151. Electroporation uses electrical pulses in the order of hundreds of volts for 10 µs–10 ms to temporarily disrupt cellular membranes 152. Due to short pulse length, electroporation largely relies upon compromising the stratum corneum to assist passive diffusion to the layers below 153, although the ability to disrupt target cell membranes also aids in the delivery of nucleic acid‐based vaccines 154. This restricts translatability when compared to a needle and syringe due to an increase in pain experienced by the patient 155, especially when used post‐injection of nucleic acid‐based vaccines. Iontophoresis, in contrast, is believed to have negligible effects on skin architecture over short treatment intervals 152. However, in 2016, Zecuity, a commercialized iontophoretic transdermal device, lost FDA approval after post‐marketing reports of application site reactions, including burns and scars in patients 156.

Sonophoresis is the use of ultrasound to improve transdermal drug and vaccine delivery 157. The main mechanism that drives the enhanced delivery of sonophoresis is cavitation 158. This process involves the application of focused ultrasound to achieve expansion and collapse of gas bubbles which, in turn, creates microstreaming and shockwaves 159. Sonophoresis has been used to either increase the permeability of the skin before topical application of the vaccine or as a method of concurrently applying both cavitation and vaccine, thereby actively pushing the vaccine particles into the skin 160, 161. The recent development of nano‐sized polymeric cavitation nuclei (nanocups), capable of sustaining and promoting cavitation activity, provides a means of sustaining such delivery over extended periods 161, 162. Indeed, when mixed with nanocups and exposed to ultrasound, the model antigen, ovalbumin, was delivered to depths of 500 µm and a specific anti‐ovalbumin antibody response was raised 161. The major limitation of sonophoresis is the inability to deliver comparable amounts of antigen to that of a needle and syringe.

Biolistics involves the use of high pressures to accelerate vaccines to velocities high enough to allow penetration of the stratum corneum and epidermal cell membranes 163. It can be used to deliver a jet of liquid or particulate vaccines 163. Jet injection of liquid vaccine was tested for smallpox vaccination 164 and has also been tested for measles, BCG and influenza 165, 166, 167, 168. In principle, nucleic acid‐based vaccines can be delivered by first coating them onto gold particles and propelling them into the skin using this approach 163. One study found that for successful delivery of particulate vaccine by biolistics, particles must be of a similar size, smaller than 70 µm, have a density above 1 g/ml and be able to maintain physical stability in the process 169. Work continues to improve the compatibility of vaccine to use in biolistic devices 170, 171, but issues remain regarding tissue damage and pain.

Transdermal delivery is an attractive goal because of the potentially easy and pain‐free access it provides to a rich immunological milieu. However, in addition to the unique limitations faced by each of the technologies described above, there is also a general challenge to overcome the huge inter‐ and intraperson hetrogeneity in stratum corneum thickness, hydration levels and hair‐follicle density. Furthermore, in order to depose needle and syringe as the preferred administration technology, all the approaches described above will also face the challenge of matching the impressive needle‐and‐syringe price point. It might be argued that patients in more economically developed countries may be prepared to pay a premium for more bespoke ‘pain‐free’ alternatives to needle and syringe. A suggestion supported by the increased cost of Flumist ($20 *versus* $15 172). However, it is clear that the ‘dollar‐per‐dose’ ideal for mass vaccination programmes in resource‐challenged countries will provide a substantial initial hurdle to widespread translation of these new approaches, especially as they also often involve bulky, complex equipment. It is difficult to compare the cost of these approaches to that of a needle and syringe in terms of vaccine delivery due to the lack of commercialization. However, in terms of drug delivery, the cost of a Zecuity patch, before the removal of its licence, was $300 per patch 173. It is therefore even more imperative that these new administration technologies show superiority in efficacy and safety.

### Mucosal administration

The majority of pathogens invade via mucosal surfaces such as the respiratory, gastrointestinal or reproductive tracts. These surfaces come into direct contact with the air, water and food from our surrounding environment, giving a first point‐of‐contact for opportunistic pathogens. Despite this fact, only five pathogens currently have mucosal vaccines licensed for their treatment: cholera, typhoid, rotavirus, poliomyelitis and influenza. Conventional systemic vaccination procedures using a needle and syringe are generally considered unable to induce strong mucosal immune responses 174. However, delivery of vaccines across mucosal surfaces has the potential to stimulate such responses, providing neutralization of invading pathogens before they are able to cause a widespread infection. Mucosal vaccination has also been shown to be able to elicit systemic immunity comparable to vaccination with a needle and syringe 175. There are, however, many challenges to mucosal vaccination, from the harsh acidic environment of the stomach to the layer of mucus which coats all mucosal surfaces. Despite these challenges, there is a wide range of mucosal vaccination routes being explored, the two most common of which are oral and intranasal; others include ocular, intravaginal and intrarectal.

Oral vaccination is a preferred route for vaccination, as it is painless, safe, low‐cost and does not require trained personnel for administration. The oral route commonly involves swallowing the vaccine which then passes through to the gastointestinal tract. Alternatively, the vaccine can also achieve entry in the oral cavity, which has far less harsh conditions 149, although this method has not been explored in the same depth. In order to induce immunity through the oral gastrointestinal route, a relatively large amount of vaccine must be administered due to factors such as dilution while passing through the gastrointestinal tract, degradation within the stomach or failure to breach the epithelial tight junctions 176. One commonly employed method of reducing the severity of the conditions in the stomach is to include a basic substance such as sodium bicarbonate to neutralize the conditions 122. There are currently oral vaccines licensed for human use for cholera (Dukoral, Vaxchora and Shanchol), poliomyelitis (OPV), rotavirus (Rotarix, RotaTeq, Rotavac, Rotavin‐M1, Lanzhou lamb and Rotasiil) and typhoid (Vivotif) vaccination.

Intranasal vaccination is a popular choice for alternative vaccination methods, as it uses a site that is easily accessible and has the potential for self‐administration. The nasal cavity is also a highly vascularized region with a large surface area for antigen uptake. The nasal route, one of the main sites of pathogen entry, thus inducing a strong local mucosal immunity, is highly desirable. As nasal vaccination delivers the antigenic material directly to the targeted site, a relatively small dose is required when compared to alternative forms of vaccination 177. However, most antigens have very little affinity for the nasal epithelium and thus have a fast clearance rate 178. Therefore, many groups have undertaken research on improving this, resulting in a wealth of patented delivery methods 179. Currently the only licensed intranasal vaccines for use in humans is for influenza (Flumist), which consists of a live attenuated virus delivered by nasal spray.

Many pathogens are transmitted sexually via the genital tract, such as HIV, HPV and chlamydia. Therefore, intravaginal vaccination has been explored as an option to prevent sexually transmitted infections 180. This is a challenging method of generating an immune response as the immunological features of the female reproductive system alter dramatically in response to hormonal fluctuations during the menstrual cycle 181. One reason this method of vaccination has not been explored as extensively as others could be that it only has the ability to immunize females in a population in which, increasingly, it is being shown that it does not provide sufficient herd immunity, especially when considering the impact of immunocompromised females who are unable to receive the vaccinations 182, 183.

Intrarectal vaccination is another method of administration explored to prevent diseases such as enteric pathogens, sexually transmitted diseases and cancer 181, 184, 185, 186. In general, an immune response is more strongly induced at the site of vaccination and in nearby mucosal sites; as such, it is possible to generate both rectal and genital tract immunity in response to an intrarectal vaccination 187. This method of vaccination is not widely used as it has poor acceptability 176.

## Conclusions

The field of vaccinology continues to advance at an impressive rate, with more effective and acceptable new vectors and approaches reaching clinical practice. The traditional ‘three Is’ model (isolate, inactivate, inject) of vaccine development is increasingly being phased out for a more rational design paradigm. Occurring alongside the progress of these new rationally designed vaccines is the development of improved and more patient acceptable delivery techniques to target and sustain the pain‐free administration of antigen more effectively. Enhancing methods for administering these vaccines is crucial in reducing the number of needle‐stick injuries and lowering the burden on medical staff by making vaccines more amenable to self‐administration. Unfortunately, new administration technologies (e.g. electroporation, sonophoresis, ionotophoresis) will never be able to match the dose level that can be delivered using a needle and syringe. However, it is hoped that by combining the new delivery methods with the raft of new more effective vaccines this limitation may be negated. Understanding the particular mechanism‐of‐action of new vaccines and vaccine delivery platforms and how their benefits can be enhanced and their limitations mitigated by matching to particular administration routes and technologies will be essential to this process. Vaccines are the world’s most effective, life‐saving, medical technology to date. The literature review presented here indicates that the range of complementary approaches and technologies emerging from research and clinical testing will allow the beneficial impact of vaccines to continue to grow. Potential barriers to the widespread uptake of these approaches will be the cost involved and the complexities of some of technology required, and so these aspects deserve careful consideration during the development phase alongside the scientific and technical challenges.

## Acknowledgements

J. W. acknowledges funding from the University of Oxford, the EPSRC and BBSRC Centre for Doctoral Training in Synthetic Biology (grant EP/L016494/1) and the Defence Science and Technology Laboratory (DSTL) (grant DSTLX‐1000102376). R. C. is supported by the EPSRC under the Oxford Centre for Drug Delivery Devices OXCD3 (Grant EP/L024012/1).

## Disclosures

The authors declare no competing interests.
